# Association of LOX-1 gene polymorphisms with cerebral infarction in northern Chinese Han population

**DOI:** 10.1186/1476-511X-13-55

**Published:** 2014-03-25

**Authors:** Xu Liu, Rui-Xia Zhu, Lei Li, Zhi-Yi He

**Affiliations:** 1 Department of Neurology, First Affiliated Hospital of China Medical University, No. 155 North Nanjing Street, Shenyang 110001, Liaoning Province, China

**Keywords:** LOX-1, Ox-LDL, Polymorphisms, Cerebral infarction, Atherosclerosis

## Abstract

**Background:**

Lectin-like oxidized low-density lipoprotein receptor-1 (LOX-1) plays an important role in the pathophysiology of atherosclerosis and thrombosis. This study is aimed at evaluating the potential association of 3’-UTR-C188T and G501C in LOX-1 gene with cerebral infarction.

**Methods:**

A total of 386 patients with cerebral infarction and 386 healthy controls were included in the study, which were unrelated Chinese Han population in the Liaoning Province of northern China. The single nucleotide polymorphisms, 3’-UTR-C188T and G501C, were analyzed by polymerase chain reaction–ligation detection reaction method.

**Results:**

The frequencies of CC + GC genotype, GC genotype and C allele of G501C in the patients with cerebral infarction were significantly higher than those in the controls (*P* < 0.01, *P* < 0.01, *P* = 0.04, respectively). The correlation still remained after adjusting for confounding risk factors of cerebral infarction. In addition, no significant association was observed between 3’-UTR-C188T and cerebral infarction.

**Conclusions:**

The study indicated that the G501C variant in LOX-1 gene may be associated with susceptibility to cerebral infarction, independent of other common risk factors, in northern Chinese Han population.

## Background

Stroke is the second most common cause of death and the leading cause of long-term disability worldwide [1]. In China, with 1.4 billion populations, the annual stroke mortality rate has exceeded heart disease to become the leading cause of death [2]. Additionally, an area of higher stroke incidence exists in the provincial regions of northern China [3]. Cerebral infarction, as the most common type of stroke, accounts for about 43.7% to 78.9% of all strokes in China [4]. Many studies have demonstrated that cerebral infarction is a heterogeneous disease with a complex etiology involving both genetic and environmental contributions [5,6].

Lectin-like oxidized low-density lipoprotein receptor-1 (LOX-1) is a trans-membrane glycoprotein which belongs to a class E scavenger receptor and mediates the uptake and internalization of ox-LDL (oxidized low-density lipoprotein) [7]. Recent studies have showed that LOX-1 may play an important role in the pathophysiology of atherosclerosis and thrombosis [8,9]. Human LOX-1 gene spans over 7000 base pairs and consists of 6 exons and 5 introns and is located on chromosome 12p12-p13 [10]. One polymorphism on the 3’-UTR of the LOX-1 gene, 188C-T, has previously been found to be associated with coronary artery disease in Italian and USA population [11-13]. Another polymorphism, a 501G-C transversion, has been found to be associated with myocardial infarction and carotid atherosclerosis [14,15]. However, up to now, little is known about whether the LOX-1 gene polymorphism is associated with the risk of cerebral infarction or not [16,17].

Therefore, in the present study, we investigated the association of these two LOX-1 gene polymorphisms (3’-UTR-C188T, G501C) with cerebral infarction in Chinese Han population.

## Results

A total of 386 cerebral infarction patients and 386 healthy controls were enrolled in this study. The characteristics of the patients and controls are shown in Table 1. There were no significant differences in age, gender, or body mass index (BMI). However, the prevalence of conventional risk factors for cerebral infarction, such as hypertension, diabetes mellitus, hyperlipidemia and smoking in the cerebral infarction patients was significantly higher than those in the controls.

**Table 1:** Clinical characteristics of study subjects

	**Cases**	**Control**	***P *** **value**
Age^a^	62.1 ± 9.89	61.9 ± 9.84	0.83
Male gender, n (%)^b^	267 (69.2)	265 (68.7)	0.88
BMI (Kg/m^2^)^c^	25.5 ± 3.25	25.2 ± 3.15	0.20
Hypertension, n (%)^b^	272 (70.5)	224 (58.0)	<0.01
Diabetes, n (%)^b^	138 (35.8)	72 (18.7)	<0.01
hyperlipidemia, n (%)^b^	173 (44.8)	136 (35.2)	<0.01
Smoking, n (%)^b^	134 (34.7)	70 (18.1)	<0.01

BMI, body mass index.

^a^Mann–Whitney *U* test is applied.

^b^χ^2^ test is applied.

^c^Student's *t*- test is applied.

The genotype and allele frequencies of two LOX-1 gene polymorphisms in patients and control subjects were shown in Tables 2 and 3. All genotype distributions in both patients and controls were in the Hardy-Weinberg equilibrium. For 3’-UTR-C188T, *P* was 0.14 and 0.86 for the patient and control groups, respectively; while for G501C, *P* was 0.17 and 0.09 for the patient and control groups, respectively. As shown in Tables 2 and 3, there was no significant difference in the distributions of genotypes and alleles of 3’-UTR-C188T between cerebral infarction patients and controls. In contrast, the significant association was observed for G501C in a dominant model. A higher frequency of the CC + GC genotype for G501C was observed in all participants with cerebral infarction (OR = 1.505, 95% CI = 1.114-2.033, *P* < 0.01). The GC genotype (35.0%) of G501C was represented at an increased frequency in the group of patients (OR = 1.596, 95% CI = 1.166-2.183, *P* < 0.01). The frequency of G501C C allele was significantly higher in patients with cerebral infarction than in the control group (OR = 1.317, 95% CI = 1.018-1.705, *P* = 0.04).

**Table 2:** Genotype and allele distributions of 3’-UTR-C188T in patients with cerebral infarction and controls

	**Cases (%)**	**Control (%)**	**OR**	**95% CI**	***P *** **value**
Genotype					
CC	229 (59.3%)	212 (54.9%)	Reference		
CT	143 (37.0%)	149 (38.6%)	0.888	0.661-1.194	0.43
TT	14 (3.6%)	25 (6.5%)	0.518	0.263-1.024	0.06
Dominant effect					
TT + CT vs CC	157/229	174/212	0.835	0.628-1.111	0.22
Recessive effect					
TT vs CT + CC	14/372	25/361	0.543	0.278-1.062	0.07
Allele					
C	601 (77.8%)	573 (74.2%)	Reference		
T	171 (22.2%)	199 (25.8%)	0.819	0.648-1.035	0.10

χ^2^ test is applied for calculation of *P* values.

**Table 3:** Genotype and allele distributions of G501C in patients with cerebral infarction and controls

	**Cases (%)**	**Control (%)**	**OR**	**95% CI**	***P *** **value**
Genotype					
GG	239 (61.9%)	274 (71.0%)	Reference		
GC	135 (35.0%)	97 (25.1%)	1.596	1.166-2.183	<0.01
CC	12 (3.1%)	15 (3.9%)	0.917	0.421-1.998	0.83
Dominant effect					
CC + GC vs GG	147/239	112/274	1.505	1.114-2.033	<0.01
Recessive effect					
CC vs GC + GG	12/374	15/371	0.794	0.367-1.718	0.56
Allele					
G	613 (79.4%)	645 (83.5%)	Reference		
C	159 (20.6%)	127 (16.5%)	1.317	1.018-1.705	0.04

χ^2^ test is applied for calculation of *P* values.

Binary logistic regression with backward stepwise method was used to evaluate the association between G501C polymorphism in LOX-1 gene and the risk of cerebral infarction in the dominant model. As shown in Table 4, logistic regression analysis revealed that the CC + GC genotype of G501C was significantly associated with an increased risk of cerebral infarction (OR = 1.520, 95% CI = 1.109-2.082, *P* < 0.01),as well as other conventional risk factors (hypertension, diabetes mellitus, smoking but not hyperlipidemia). In order to evaluate the goodness-of-fit of the model, we have used the Hosmer-Lemeshow test. The model correctly predicted 61.7% of the cases and the Hosmer-Lemeshow goodness-of-fit test demonstrated that the calibration of the model was satisfactory (χ^2^ = 9.747, 8df, *P* = 0.283).

**Table 4:** Cerebral infarction risk factors and G501C in the logistic regression analysis

	**Adjusted OR**	**95% CI**	***P *** **value**
hypertension	1.522	1.112-2.081	<0.01
Diabetes	2.305	1.633-3.254	<0.01
Smoking	2.418	1.717-3.407	<0.01
CC + GC vs GG	1.520	1.109-2.082	<0.01

## Discussion

This study investigated the association between LOX-1 gene polymorphisms, 3’-UTR-C188T and G501C, and cerebral infarction in northern Chinese Han population. In this study, we found that the genotypic and allelic frequencies of G501C were significantly associated with the increased risk of cerebral infarction. However, no association of 3’-UTR-C188T with the risk of cerebral infarction was found.

The 3’-UTR-188 T variant may have effects on transcription at the gene level and affect the exon splicing or the binding affinity of a putative regulatory element [13]. Therefore, the studies on different populations have been performed to uncover whether 3’-UTR-C188T in LOX-1 gene could participate in cardiovascular disease. Mango and Noveli [11,12] found that there was significant association between 3’-UTR-C188T variant and coronary artery disease in Italian population, indicating that carrier of allele T increased the risk of cardiovascular events, which was consistent to USA population as reported by Chen [13]. Nevertheless, this association has not been replicated in Italian population by Trabetti or Sentinelli [18,19]. In this study, for the first time, we focus our attention on the association between 3’-UTR-C188T in LOX-1 gene and cerebral infarction. We found that the TT + CT genotype, the TT genotype, the CT genotype and T allele of 3’-UTR-C188T were not associated with the increased risk of cerebral infarction in Chinese Han population.

As for G501C variant in LOX-1 gene, its association with cardiovascular disease is also quite contradictory. Tatsuguchi et al. [14] found the significant association of G501C variant with myocardial infarction in Japanese population, which was opposite to Turkish population as reported by Kurnaz [20]. As for cerebral infarction, Hattori et al. [17] found that the CC + GC genotype and the C allele were not associated with cerebral infarction in Japanese population (*P* = 0.48, *P* = 0.91). However, in this study, we found that the CC + GC genotype, the GC genotype and C allele of G501C were significantly associated with the increased risk of cerebral infarction in Chinese Han population. After adjusting for the confounding risk factors, this significant correlation still remained. There are two potential explanations for the divergent results. Genetic heterogeneity may be the important reason. Genotype frequencies of the G501C in our control subjects (71% GG, 25% GC, and 4% CC) are different from those in Japanese population (61% GG, 36% GC, and 3% CC). In addition, the relative small sample size of Hattori’s study, consisting of 235 patients with cerebral infarction and 274 controls, might be more likely to get false negative results.

How can we explain the association between G501C in LOX-1 gene and cerebral infarction in Chinese population? 1) LOX-1 is involved in endothelial activation and dysfunction, monocyte adhesion, the proliferation, migration, and apoptosis of smooth muscle cells, foam cell formation, plaque instability, as well as platelet activation; all of these events are critical in the pathogenesis of atherosclerosis and thrombosis [9,21]. 2) The G501C polymorphism is a coding region variant identified in the LOX-1 gene, which causes an amino acidic substitution (lysine to asparagine at position 167 in the C-terminal domain). Functional analysis has demonstrated that the substitution reduces ox-LDL binding and uptake in vitro. This nonsynonymous change also alters the ox-LDL -induced LOX-1 expression [22].

## Conclusions

In summary, our study demonstrated that G501C, but not 3’-UTR-C188T, in LOX-1 gene may be associated with susceptibility to cerebral infarction, independent of other common risk factors, in northern Chinese Han population. However, the present study also has some limitations, such as the relative small sample size. Therefore, well-designed studies with large sample sizes regarding the association of LOX-1 gene polymorphisms with cerebral infarction on different ethnic population will be needed to verify these findings in the future.

## Methods

### Subjects

The present study included 386 cerebral infarction patients and 386 healthy controls, which were unrelated Chinese Han population in the Liaoning Province of northern China. The patients were consecutively hospitalized in the Department of Neurology, the First Affiliated Hospital of China Medical University between September 2011 and December 2012. Eligible patients were defined as those who were first diagnosed with acute cerebral infarction according to neurological examination and radiological imaging. According to TOAST classification, cerebral infarction can be divided into five subtypes: 1) large-artery atherosclerosis (LAA), 2) small-vessel occlusion (SVO), 3) cardioembolism (CE), 4) stroke of other determined etiology, and 5) stroke of undetermined etiology [23]. Patients with LAA and SVO, two most common subtypes of cerebral infarction, were included while other subtypes were excluded. Patients with transient ischemic attack, cerebral trauma, cerebrovascular malformations, coagulation disorders, autoimmune diseases, tumors, and chronic infection diseases were all intentionally excluded from this study. The controls were recruited from the health examination department of the Red Cross Hospital, matched by sex and age, without clinical or radiological evidence of stroke and other neurological diseases. This study was performed according to Declaration of Helsinki and the standards established by the Ethics Committees of both hospitals. The Ethics Committees of both hospitals reviewed and approved the study protocol, and written informed consents for the study were obtained from all participants.

Clinical records of patients and controls, including the general condition, past history, blood pressure, fasting blood glucose, serum triglyceride, and total cholesterol, were collected. For patients, brain CT or MRI, echocardiography, carotid ultrasound, transcranial Doppler, and electrocardiogram were also required. Hyperlipidemia was defined as a total plasma cholesterol level >5.72 mmol/L and/or plasma triglycerides level >1.7 mmol/L, or current use of lipid-lowering drugs.

### DNA Extraction and Genotyping

Genomic DNA was extracted from EDTA-anticoagulated peripheral blood by a DNA Purification Kit (Promega, Madison, USA). Genotypings were determined using the polymerase chain reaction-ligation detection reaction (PCR-LDR) method [24]. DNA purity was assessed by spectrophotometry, and DNA samples were stored at -20°C. The sequences of primers for PCR are shown in Table 5. PCR amplification was performed in a total volume of 10 μL that contained 1× GC buffer I (Takara, Japan), 3.0 mM Mg^2+^ (Takara, Japan), 0.3 mM dNTP (Generay Biotech, China), 1 U HotStarTaq polymerase (Qiagen, Germany), 1 μL each primers (Sangon, China) and 1 μL genomic DNA. The PCR cycling program was set at 95°C for 2 min, followed by 11 cycles of 94°C for 20 s, 65°C (decreased 0.5°C per cycle) for 40 s, 72°C for 1.5 min, and then 24 cycles of 94°C for 20 s, 59°C for 30 s, and 72°C for 1.5 min, and a final extension at 72°C for 2 min. Then shrimp alkaline phosphatase (Promega, USA) and Exonuclease I (Epicenter, USA) were added into the PCR products for purification. The probes for LDR are shown in Table 6. The LDR reactions for each PCR product were performed in a final volume of 10 μL, containing 1 μL 10× ligase reaction buffer (New England Biolabs, USA), 2 μL purified PCR product, 0.4 μL 5’ ligase primer (1 μM) mixture (Sangon, China), 0.4 μL 3’ ligase primer (2 μM) mixture (Sangon, China), 0.25 μL Taq DNA ligase (New England Biolabs, USA) and 6 μL double distilled H_2_O. The LDR reactions were cycled as: 38 cycles of 94°C for 60 s and 56°C for 4 min, and kept at 4°C. After the reaction, 0.5 μL LDR product was then sequenced with ABI3730XL sequencer (Applied Biosystems, USA). Finally the raw data was analyzed by GeneMapper 4.1 (Applied Biosystems, USA).

**Table 5:** Primer sequences of 3’-UTR-C188T and G501C

**SNPs**	**Primer sequences**
3’-UTR-C188T	Forward: 5’ TTAGGAGTGTGAGGGGAAGGTGA 3’
	Reverse: 5’ CCTTTGCAGAAACTGGGGTTCC 3’
G501C	Forward: 5’ TGTCCGTCCAAGGTCATACACAA 3’
	Reverse: 5’ CCTTGTCCGCAAGACTGGATCT 3’

**Table 6:** The probes for LDR

**SNPs**	**The probe for LDR**
3’-UTR-C188T	FA: TGTTCGTGGGCCGGATTAGTGCTTGGGACAAGCTAGGTGAAATAATACTGA
	FG:TCTCTCGGGTCAATTCGTCCTTGCTTGGGACAAGCTAGGTGAAATAATACTGG
	FP: TAGCTAGAATCAAAAATGTTGACATAAAGGTGTTTTTTTTTTTTTTTTTTTT
G501C	RC: TCTCTCGGGTCAATTCGTCCTTTCGGGCTCATTTAACTGGGAACAG
	RG: TGTTCGTGGGCCGGATTAGTTCGGGCTCATTTAACTGGGAACAC
	RP: AGCCAAGAGAAGTGCTTGTCTTTGTTTTTTTTTTTTTT

LDR, ligation detection reaction.

### Statistical analysis

Continuous variables were presented as mean ± SD and categorical variables as percentages. Normality of the sample distribution of each continuous variable was tested with the Kolmogorov-Smirnov test. Differences of continuous variables were evaluated by the Student's *t* or Mann–Whitney *U* test, depending on the shape of the distribution curves. Categorical variables were compared by *χ*^2^ test. Differences of the distributions of alleles and genotypes between cases and controls were analyzed using *χ*^2^ test. All genotype frequencies were checked for Hardy-Weinberg analysis in two groups through *χ*^2^ test [25]. The association of the LOX-1 gene polymorphisms with cerebral infarction was estimated by computing the odds ratios (OR) and 95% confidence intervals (CI) from binary logistic regression analysis with backward stepwise method. Furthermore, Hosmer-Lemeshow test was used to evaluate the goodness-of-fit of the logistic regression model. A *P* value of 0.05 was considered statistically significant for all statistical analyses. All the statistical analyses were carried out using SPSS version 17.0 (SPSS, Chicago, IL, USA).

## Abbreviations

LOX-1: Lectin-like oxidized low-density lipoprotein receptor-1; ox-LDL: Oxidized low-density lipoprotein; PCR-LDR: Polymerase chain reaction-ligation detection reaction; OR: Odds ratio; CI: Confidence interval; BMI: Body mass index.

## Competing interests

The authors declare that they have no competing interests.

## Authors’ contributions

XL carried out the molecular genetic studies and drafted the manuscript. XL and RXZ carried out the genotyping. XL and LL participated in the design of the study and performed the statistical analysis. ZYH and XL conceived of the study, and participated in its design and coordination. ZYH helped to draft the manuscript. All authors read and approved the final manuscript.
